# Current Methods for Identifying Plasma Membrane Proteins as Cancer Biomarkers

**DOI:** 10.3390/membranes13040409

**Published:** 2023-04-05

**Authors:** Edwin de Jong, Armagan Kocer

**Affiliations:** Department of Bioelectric Signaling and Engineering, Faculty of Science and Technology, University of Twente, Enschede 7500 AE, The Netherlands; e.dejong@utwente.nl

**Keywords:** biomarkers, cancer, membrane proteins, cell surface profiling, tumor markers

## Abstract

Plasma membrane proteins are a special class of biomolecules present on the cellular membrane. They provide the transport of ions, small molecules, and water in response to internal and external signals, define a cell’s immunological identity, and facilitate intra- and intercellular communication. Since they are vital to almost all cellular functions, their mutants, or aberrant expression is linked to many diseases, including cancer, where they are a part of cancer cell-specific molecular signatures and phenotypes. In addition, their surface-exposed domains make them exciting biomarkers for targeting by imaging agents and drugs. This review looks at the challenges in identifying cancer-related cell membrane proteins and the current methodologies that solve most of the challenges. We classified the methodologies as biased, i.e., search cells for the presence of already known membrane proteins. Second, we discuss the unbiased methods that can identify proteins without prior knowledge of what they are. Finally, we discuss the potential impact of membrane proteins on the early detection and treatment of cancer.

## 1. Introduction

**Figure 1:**
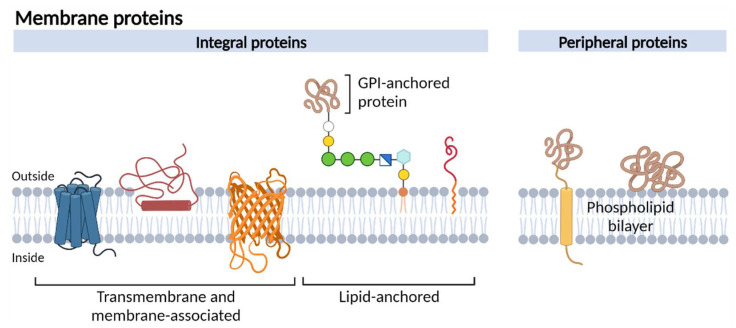
Schematic representation of different membrane protein types.

In every cell, a plasma membrane made up of a phospholipid bilayer separates the cells’ content from the outside. More than 50% of this membrane is decorated with special proteins that can be associated with the inner or outer side of the membrane or traverse the entire bilayer [1,2,3] (Figure 1). While the cell membrane provides an almost impenetrable surface for charged molecules, plasma membrane proteins facilitate well-regulated traffic of ions, water, or nutrients across the membrane, define the immunological identity of the cell, enable communication with the extracellular matrix, and participate in communication between the inside and outside of the cell [4]. Thanks to their unique features, membrane proteins have high potential as biomarkers. First, 30% of all human genome encodes for the different membrane proteins involved in almost all physiological processes [5]. Hence, their aberrant expression, differential modification, changed interacting partners, or malfunctioning is linked to many important diseases, including cancer [1,2,3,6]. Second, the extracellular domains of membrane proteins at the cells’ surface expose them to ready detection with probes and antibodies, or marking for imaging of the diseased tissue, for diagnosis, prognosis, or visualizing tumor boundaries to enhance precise dissection in surgery [7]. Third, they may also simultaneously serve diagnosis, imaging, and targeting purposes. For instance, human epidermal growth factor receptor 2 (HER2 or CD340) and epidermal growth factor receptor (EGFR) are readily accessible cell surface markers and specific docking sites for toxic drug-conjugated monoclonal antibodies. They facilitate high specificity and low toxicity treatment [8,9,10].

Historically, plasma membrane protein’s potential role as biomarkers has roots since the 1970s. Davey et al. recognized their involvement in cancer in the context of metastasis, i.e., the spreading of the primary tumor to other body parts. They proposed that neither a single determinant on the membrane but rather the cell membrane and its proteins as a whole might be related to metastasis [11]. The membrane vesicles, now known as extracellular vesicles, which are shed from highly metastatic B16 mouse melanoma cells (F10 subline) in culture, could fuse to poorly metastatic cells (F1 subline) and convert them into metastatic phenotype. However, membrane vesicles from other non-metastatic B16 sublines did not modify the metastatic capacity of F1 cells. Since membrane vesicles originated from the plasma membrane of the donor cell, the results indicated that differences in cell surface properties determine the differences in the metastatic behavior of F1 and F10 cell lines [11].

More direct evidence for plasma membrane proteins’ involvement in cancer became apparent when researchers observed the expression of unusual patterns of functional ion channels, a class of membrane proteins, in cancer cell lines [12]. Furthermore, showing the inhibitory effect of a targeted blockage of such ion channels on cancer growth made it clear that membrane proteins have significant roles in cancer pathophysiology and can be valuable targets for therapy. Since then, efforts have been made to identify the cell surface proteins differentially expressed in cancer or their surrounding cells for diagnostics and therapy.

However, challenging physicochemical properties of membrane proteins have markedly restrained research efforts. Their transmembrane segments are hydrophobic, limiting their solubility in aqueous environments and complicating their purification and identification with conventional techniques [13]. In addition, they are heavily outnumbered by intracellular soluble proteins, a challenge that requires instrumentation with sufficient resolving power and sensitivity to detect low-abundant species [14]. Finally, the repertoire of membrane proteins on the cell membrane is very dynamic [15,16] due to the secretion, internalization, and shedding of proteins in response to internal and external signals [17]. As a result, for a long time, techniques for the direct detection of membrane proteins were either inadequate or lacked ease of use. However, with recent technological developments, profiles of cancer cell surface proteins are emerging rapidly.

This review focuses on the current methods for identifying the cell surface membrane proteins as cancer biomarkers. However, it is worth noting that membrane proteins in other membrane-bound compartments of the cell, such as mitochondria, endoplasmic reticulum, and lysosomes, are also related to the hallmarks of cancer and are proposed as potential organelle-targeted cancer therapies [18]. These proteins are involved in regulating the tumor cell metabolism and fate by facilitating dynamic information exchange between the cellular organelles during oncogenesis. Specifically, organelle membrane proteins are involved in forming membrane contact sides (MCS) between different organelles. These dynamic platforms facilitate the direct exchange of macromolecules between the organelles and recruit machinery for regulating physiological functions, such as apoptosis, calcium signaling, and lipid signaling, which have vital roles in tumor pathophysiology. Comprehensive reviews on this topic can be found in the following references [18,19,20].

## 2. Identifying Membrane Proteins as Cancer Biomarkers

### 2.1. Indirect Discovery Methods

The classical view of the biological information flow, known as the central dogma in molecular biology, states that DNA codes for RNA, which codes for proteins [21]. Indeed, initial attempts to identify membrane proteins have been based on predicting their identity, structure, and even function, based on gene sequences by employing bioinformatics tools [22,23,24]. However, it became clear that the linear nucleic acid sequence in the gene does not directly correlate with the protein expression, structural information, or function, and not all membrane proteins are on the cell surface [23,24,25].

In the context of cancer, Sun et al. took advantage of the genomic changes in tumor cells to identify membrane proteins through screening genes [26]. They aimed to identify ion channel prognostic signatures for head and neck squamous cell carcinoma (NHSCC) [27] upon investigating the ion channel mutations under mutations in the TP53 gene. The rationale of their study was that the most common genetic mutations in this type of cancer occur in the TP53 gene, which encodes for p53 protein. The wild-type p53 is a tumor suppressor and cells with mutated p53 are prone to develop cancer. Since ion channels are also involved in all cancer hallmarks, they argued that cells with TP53 gene mutations might alter the expression of cancer-related ion channel genes. Thus, upon a systematic investigation of aberrant ion channel genes under TP53 mutation in NHSCC and through a series of machine learning algorithms, they established seven ion channel genes as this cancer’s ion channel diagnostic signature [26,27].

In an mRNA-based approach, Diehn et al. employed a genome-scale method [28] to classify thousands of human gene products into membrane-associated or shed versus cytosolic/nuclear proteins. The procedure is based on the subcellular location of translation, i.e., synthesis of protein from mRNA, of these protein types. While membrane protein-encoding mRNAs are translated by polyribosomes attached to the cytoplasmic surface of the endoplasmic reticulum, mRNAs encoding soluble or nuclear proteins were processed by polyribosomes in the cytoplasm. This spatial preference allows the separation of proteins into membrane-associated and free mRNA fractions by equilibrium density-gradient centrifugation. Next, each mRNA fraction was differentially labeled with a fluorescent dye and analyzed using cDNA microarrays to identify the encoded proteins. After validating the approach by comparing the results to that of in silico algorithms and curated/published data sets, they set out to identify membrane-associated tissue- and tumor-specific markers [29]. By comparing their data with data obtained from the gene expression profiles of tumor samples and normal tissues, the authors could identify hundreds of genes encoding for membrane proteins.

**Figure 2:**
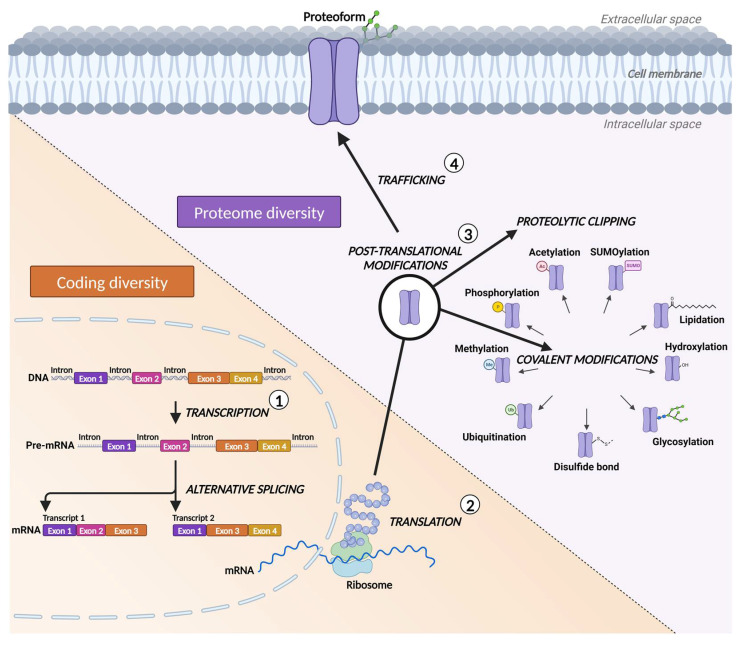
Coding and proteome diversity generate biological variation beyond the predictive power of initial DNA and mRNA sequences of encoded proteins through alternative splicing of mRNA and dynamic post-translational modifications of the proteins.

However, predicting the global membrane proteome from genomics and mRNA transcriptomics data is impeded by mechanisms that introduce further diversities to the genetic code at the mRNA and protein levels [30] (Figure 2). The genetic code is expanded at the transcriptional level when an RNA copy of the DNA is made by alternative splicing (or mis-splicing in pathological conditions) of mRNA, which generates multiple distinct isoforms of a protein [30,31]. After mRNA is translated into a protein, post-translational modifications in the form of covalent addition of functional groups to the protein or its proteolytic clipping introduce the next level of enrichment to the initial code [30]. Thus, the identification of clinically relevant membrane proteins’ proteoforms requires more direct approaches [32].

### 2.2. Direct, Biased Discovery Methods

#### 2.2.1. Multiplexed Immunohistochemistry/Immunofluorescence

Before the advent of the current mass spectrometry methods, affinity-based approaches, such as immunohistochemistry [33], played a significant role in identifying biomarkers in formalin-fixed, paraffin-embedded (FFPE) tissue slices by detecting the presence of monoclonal antibodies against known proteins [34]. While also providing spatial information, which might give insights into the biomarker involvement in the pathogenic mechanism, a few factors have limited biomarker detection by conventional immunohistochemistry. What could be detected was biased and dependent on the availability and the quality of the specific antibodies. The number of independently detectable reporter signals was a bottleneck for simultaneously identifying multiple markers in a tissue section. Another critical challenge was the inter-observer variability inherent to manual microscopic examination [33]. However, over the years, immunohistochemistry has advanced to a highly multiplexed quantitative single-cell analysis method, i.e., multiplexed immunohistochemistry/immunofluorescence (mIHC/IF) [33], with the ability to detect 100 different markers at once when combined with digital image analysis [35]. As a result, even though the detection is still biased for known proteins, many cancer markers with prognostic and predictive values could be identified [35]. For instance, Toh et al. employed this method to profile gastric cell surface markers for discriminating between normal and tumor gastric cells [36]. Previously, several genes and proteins were shown to be differentially expressed in gastric cancer; however, a systematic study on the cell surface markers was missing. The authors opted to profile the cell surface and generate a panel of surface proteins to differentiate tumor cells from normal cells. They first used the whole transcriptome sequencing of gastric cancer, matched normal cells, and identified a cohort of upregulated putative cell surface proteins. Then, by using a tissue microarray of normal, marginal, and tumor tissues, they performed mIHC/IF and digital image analysis using antibodies to the candidate membrane proteins from the cohort. They could identify the cell adhesion molecules CEAMCAM5, CEAMCAM6, EpCAM, and a tumor-associated glycoprotein CA 72-4 as cell surface markers, which can be used to identify tumor tissue in gastric cancer [36].

#### 2.2.2. High-Throughput Flow Cytometry

An alternative to conventional immunohistochemistry for identifying cell surface proteins is flow cytometry (FC). Similar to immunohistochemistry, FC requires reporter-tagged monoclonal antibodies to assess the presence or absence of known surface proteins. In a typical workflow, the surface proteins on intact cells are stained with fluorescent probes, after which the cells are washed and introduced into the fluidic system. Cells are aligned in a single file and pass individually through a laser beam, after which fluorescent light is detected in multiple channels to infer the abundance of corresponding membrane proteins [37]. While the conventional FC can detect up to 11 different proteins at a time, the current high-throughput flow cytometry (HT-FC) is able to detect hundreds of different surface proteins in every single cell in a cell suspension. Gedye et al., for instance, combined a robust and rapid FC platform with a high-speed automated sample loading device to screen tumor cells for the presence of known surface protein markers using hundreds of unique antibodies targeting cell surface proteins [38]. In analogy to gene expression microarrays, they arrayed 363 fluorochrome-conjugated surface-protein-targeting antibodies into several 96-well plates, one antibody per well. After proving the reproducibility and feasibility of the method using tumor cell lines, they applied the HT-FC to test primary tumor tissues that have a heterogeneous mixture of different cell types. To cope with such a complex mixture of infiltrated immune cells, stromal cells, and tumor cells, they prepared a single-cell suspension of the tissue. They analyzed it in the protein array with up to five additional antibodies per well for co-staining cells with cell type- or cell lineage-specific markers. Based on supervised hierarchical clustering, they could reveal several antigen clusters that delineate the analyzed cell lineages and identify previously unknown markers for a given cell lineage [38]. In another study, the HT-FC screening platform was used by Chen et al. to investigate the expression of a cluster of differentiation (CD) antigens on patient-derived hepatocellular carcinoma cells [39].

### 2.3. Direct, Unbiased Discovery Methods

#### 2.3.1. Bottom-Up Mass Spectrometry

The most commonly used method for unbiased identification of protein biomarkers is bottom-up mass spectrometry [40,41,42], where proteins are enzymatically or chemically digested, and the resulting peptides are sequenced by the mass spectrometer. The peptide sequence information is used to reconstruct the identity of the parent protein using databases. Mass spectrometry (MS) methods do not rely on antibodies and can directly detect thousands of proteins or peptides in a single experiment without any label. However, low abundance, poor solubility in aqueous solvents, and the limited number of enzymatic cleavage sites of membrane proteins demand several modifications to the common MS workflows [41]. This section discusses how these modifications enable quantitative analysis of membrane proteins and their proteoforms for cancer biomarker discovery.

##### Enrichment

Soluble intracellular proteins are highly abundant and tend to hinder the detection of plasma membrane proteins in complex samples. Since the sensitivity of MS is low for detecting molecules with concentrations below 50 pg/mL, MS analysis of membrane proteins is often preceded by an enrichment procedure [14,43]. Historically, plasma membranes were retrieved by separating subcellular fractions through density-gradient or differential centrifugation methods as early as the 1970s [44]. However, these techniques require high sample loads and often suffer from significant cross-contamination from other cellular compartments, and may cause false positives [16,45]. Various washing strategies have been applied to remove the contaminants. The rate of membrane protein identification varied between 20 to 60% [46].

A comprehensive review of the detailed enrichment strategies can be found in Li et al. [44]. Here, we highlight cell surface capture (CSC) [47]. In the first step of this tandem affinity labeling methodology, a wide variety of bi-functional chemical molecules are used to attach an affinity tag to the cell surface proteins by targeting either their glycan structures, primary amines, sulfhydryl residues, or carboxyl groups [14,48,49,50]. After the surface-tagged cells are homogenized and proteins are digested, the peptides bound to the affinity tag are captured through affinity purification, enzymatically released from the tag, analyzed by liquid chromatography with tandem mass spectrometry (LC–MS–MS), and the proteins are then identified [47].

While CSC targeting the primary amines or lysine groups reached moderate enrichment of membrane proteins, CSC targeting N-glycoproteins preferentially reached 90% enrichment [51]. Variations in the CSC technology also apply to primary cells and live single-cell suspensions of tissues and organs. As glycoproteins are upregulated in numerous cancers [52], this methodology has been successfully employed to identify tumor-specific glycoproteins, such as CA 125 for ovarian cancer, CA 19-9 for gastrointestinal and pancreatic cancer [44], or many differentially expressed glycoproteins associated with aggressiveness in prostate cancer [53,54,55]. Recently, the Wollscheid group miniaturized and automatized the CSC technology to reduce the initial amount of cells and minimize the sample loss [56]. This auto-CSC enhanced the sensitivity and reproducibility of quantitation and could discriminate cancer cell lines based on their surface glycoproteome [56]. Thanks to the success of the CSC methodology and the amount of generated knowledge, a mass-spectrometry-derived Cell Surface Protein Atlas (CSPA), providing cellular surfaceome snapshots at high resolution, was built [57].

##### Solubility

The typical mass spectrometry workflow includes the separation of proteins, followed by their detection. In a conventional approach, proteins are separated on a two-dimensional gel based on their isoelectric point and molecular mass, followed by matrix-assisted laser desorption/ionization mass spectrometry (MALDI-TOF-MS) [58]. In the case of membrane proteins, while their extra- and intracellular parts are hydrophilic, a significant portion of them embedded in the membrane is hydrophobic, which is responsible for the tendency of membrane proteins to precipitate at their isoelectric points. Thus, such membrane proteins are already lost from the analysis in the first dimension of the separation [59].

That is why another approach, i.e., liquid chromatography with tandem mass spectrometry (LC–MS–MS), is preferred for membrane protein research, as it eliminates the need for keeping hydrophobic proteins in solution [13]. Proteins are first digested by enzymatic shaving, and the resulting fragments are separated by liquid chromatography and sprayed directly into the mass spectrometer. During enzymatic shaving, the intact cells are washed to remove weakly bound proteins and then treated with proteases to cleave the protruding extracellular domains [60]. Due to the solubility of these ‘shaved’ fragments, they can be readily analyzed by mass spectrometry. However, the coverage depends on the accessibility of the cleavage sites, which are often compromised by protein glycosylation or folded configurations [60]. Additionally, potentially valuable information about the transmembrane region is discarded.

A solution for the challenges caused by the hydrophobicity of membrane proteins in their identification is to solubilize the membrane-embedded part of these proteins. Amphipathic molecules, such as chaotropes, organic solvents, and detergents, disintegrate the membrane and solubilize the membrane proteins to various extents. The type of solvent should be carefully evaluated, as a bias on the solubilization target might result in identifying a subpopulation rather than the whole cell membrane proteome. Comprehensive reviews on the detergent and organic solvents used for protein fragmentation and the recent developments in membrane protein digestion methods can be found in Vit and Petrak [46] and Zhang [61].

Once the membrane fraction is solubilized, the solvent is removed from the samples before digestion and MS analysis using numerous methods, including extraction methods, gel-based separation, and the covalent capture of proteins by magnetic beads. Alternatively, filter-assisted sample preparation (FASP) methods can be employed for the cleaning and digestion of samples for MS [62,63,64,65]. FASP removes not only the detergents but also chaotropes, lipids, salts, and nucleic acids. Integrating FASP methods into proteomics also boosted membrane protein identification. Combined with SDS solubilization, FASP allows the identification of thousands of membrane proteins in a single study. For instance, by incorporating FASP into the MS process, Yu et al. identified more than two thousand integral membrane proteins from human leukemia cells [66]. At the same time, Raimondo found 300 in human renal carcinoma [67].

#### 2.3.2. Top-Down Mass Spectrometry

Unlike the peptide-based bottom-up approach, top-down mass spectrometry analyzes intact proteins. It directly provides information on the proteoforms and membrane–protein interaction partners, annular lipids, and stoichiometry [68,69,70,71]. In addition, as shown by Delcourt et al., the truncated forms of proteins originating from alternative or novel open reading frames can also be identified [72]. Such so-called alternative proteins may remain hidden in proteomics studies using conventional databases due to an arbitrary length threshold to identify open reading frames [73]. Delcourt et al. aimed to characterize specific protein profiles in different regions of ovarian tumors. By employing top-down tissue microproteomics and mass spectrometry imaging, they identified 15 unknown alternative proteins that were differentially expressed in the benign, necrotic, or tumor regions of the patient biopsies [72], which may have otherwise remained undiscovered for diagnostic and therapeutic opportunities. In a systematic RNA-Seq investigation, Erady et al. identified the transcript encoding novel open reading frames and their protein products in 22 cancer types [73]. They showed that many such proteins are often expressed in multiple cancers, and some are unique to specific cancer types and can be targeted for therapeutic purposes. While gradually developing on the technical [74,75,76,77,78] and data analysis fronts [79,80,81,82], the application of top-down proteomics for cancer cell profiling is yet to be seen.

### 2.4. Contemporary Methods

#### 2.4.1. Mass Cytometry

Mass cytometry, or cytometry by time-of-flight (CyTOF), is a method to identify phenotypes of cells in the tissue, blood, or suspension in real time based on their surface or cytoplasmic protein profiles [83]. In this quantitative and highly parametric methodology, dozens of markers can be detected simultaneously by high-frequency laser ablation and time-of-flight. As in flow cytometry, target proteins are detected by target-specific antibodies. However, CyTOF antibodies are labeled with rare heavy metal isotopes with no spectral overlap instead of fluorochromes. The labeled sample is applied to a microfluidic system coupled to a nebulizer to spray single cells into inductively coupled argon plasma, which vaporizes the cells and induces ionization [83,84]. A quadrupole mass analyzer removes biological elements in the resulting ion cloud, and enriched metal reporter ions are measured by time-of-flight (TOF) mass spectrometry and integrated per individual cell as a proxy for the abundance of corresponding markers. Each metal’s mass is identified as a discrete value and exhibits minimal signal overlap with others. Currently, CyTOF allows the detection of 60 different markers simultaneously in each cell in the sample [85]. Since it allows broad-scale cell profiling, the required amount of sample is also significantly less than flow cytometry, making it a useful tool for analyzing small samples, such as children’s tumors and biopsies [86]. CyTOF relates tumor pathology to different cellular signaling pathways, evaluates the immune response to disease, and may guide the therapy. One drawback of the method is that, since the cells are evaporated during the process, they cannot be used for downstream analysis [87,88]. Another initial challenge was the CyTOF reporters’ sensitivity to detecting very low-abundant membrane proteins. However, the newest mass spectrometers overcame this challenge. The field aims to reach the theoretical 120-parameter per cell per time limit [88,89]. CyTOF has been successfully used for immunophenotyping, immuno-oncology, and oncology and has been adopted for use in clinical trials.

#### 2.4.2. Cell-SELEX

**Figure 3:**
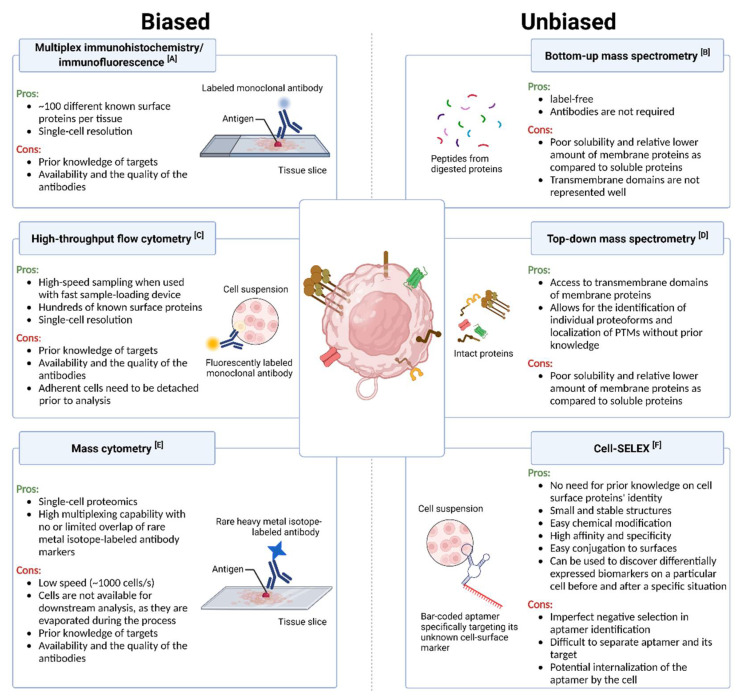
Comparison of main tools for plasma membrane identification. Biased techniques are based on known surface proteins and a labeled recognition element to detect them. Unbiased methods do not require prior knowledge of the protein’s identity, and they are label-free. References as follows: for panel A [33,34], panel B [41,42], panel C [37,38,108], panel D [40,68,69,71,73,75], panel E [84,87,89,109,110] and panel F [43,111,112].

Cell-SELEX, the Systematic Evolution of Ligands by Exponential Enrichment, is an aptamer-based tool. Aptamers are short, single-stranded synthetic DNA or RNA molecules that can selectively bind to their targets, including proteins, peptides, and carbohydrates. Cell-SELEX adopts aptamers for screening the whole cell directly for its surface proteins without knowing what those proteins are. The selected aptamers are against the native form of the surface proteins and, hence, are directly relevant to cancer cell surface profiling [90]. In this procedure, the target cells are incubated with the starting pool of a single-stranded DNA or RNA library. After the oligonucleotides are bound to the positive cells, they are eluted by heat (positive selection) and are incubated—this time with the negative cells. Next, the unbound oligonucleotides from the negative cells (negative selection) were amplified by PCR to generate a new library for the next round of positive and negative selections, as before. At the end of about twenty selection cycles, aptamers are developed. After cloning and sequencing, the aptamers for a given cell type are obtained [90]. This strategy has been successfully applied to identify cancer cell surface proteins, especially when the tumor is aggressive and heterogeneous with no known surface markers [91,92]. Shangguan et al. successfully applied the Cell-SELEX method and identified the cell-specific surface proteins in tumor cells [91]. They first found five aptamers, with very low K_D_ values ranging from 0.8 to 30 nM, against acute lymphoblastic leukemia (ALL) in cultured cells. These aptamers could then differentially identify different leukemia cells [91,93]. Then, they used one of the aptamers as bait, and through aptamer-based affinity purification and mass spectrometry analysis, they identified its target of one as a transmembrane receptor tyrosine kinase 7 (PTK7) [94]. In 2020, the same aptamer was used as a theranostic agent for hemato-oncological malignancies by Sicco et al. [95]. Other examples include the identification of differentially expressed membrane proteins of nasopharyngeal carcinoma cells [96] and pancreatic cancer cells [92], discriminating high-metastatic from low-metastatic cancer cells in colorectal carcinoma [97], breast cancer [98,99], osteosarcoma [100], prostate cancer [101,102], hepatocellular carcinoma [103,104,105], and colon cancer [106,107]. An in-depth review of the aptamer-based biomarker detection platforms and their application to various other diseases can be found in Huang et al. and Shigdar et al. [43,92]. A summary of the pros and cons of the biased and unbiased plasma membrane detection methods can be seen in Figure 3.

## 3. Conclusions and Future Perspectives

This review provides insights into some of the most promising technologies to identify membrane proteins involved in cancer. It explains the main challenges in their identification, current solutions, and the impact of such knowledge on cancer.

Cancer is one of the leading causes of death in the world, with an increasing number of incidences annually [113]. However, almost half of the cases are detected at a late stage when most cancer cells are already resistant to therapy and metastasize to other body parts, which limits the treatment options and survival rates. In a recent review, Crosby et al. identified five main challenges to be solved for detecting cancer at an early and treatable stage [114]. These are understanding the biology and behavior of the early disease, determining the risk of developing cancer, finding and validating early cancer biomarkers, developing technological tools to detect early biological changes, and evaluating and validating these tools in clinical trials.

**Figure 4:**
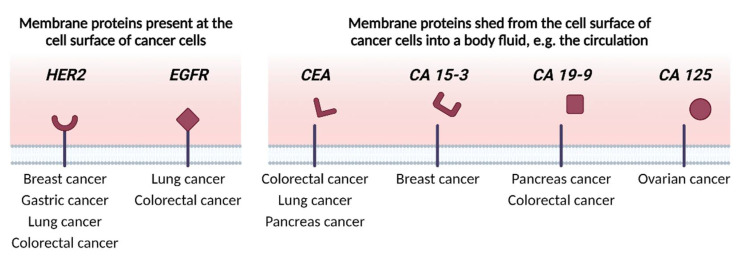
Examples of clinically used membrane proteins for the diagnosis, prognosis and/or treatment of different types of human cancer. Human epidermal growth factor receptor 2 (HER2) [115,116,117,118,119,120,121,122] and epidermal growth factor receptor (EGFR) [123,124,125,126] represent examples of membrane proteins that are present at the cell surface of specific cancer cells. Carcinoembryonic antigen (CEA) [127,128,129,130,131,132], cancer antigen 15-3 (CA 15-3) [133,134], cancer antigen 19-9 (CA 19-9) [127,131,135,136], and cancer antigen 125 (CA 125) [137,138] are (part of) the membrane proteins that are shed from the cell surface of cancer cells into a body fluid.

To this end, being needed in all cellular (mal-)functions and cells’ adaptation to changes, membrane proteins and their soluble ectodomains shed from the membrane surface into body fluids offer great opportunities for membrane protein research to contribute to the solution of most of these problems. Figure 4 shows, based on extensive clinical data, the membrane proteins that have already entered the clinics for routine use in the diagnosis, as well as the prognosis and treatment of several cancers.

**Figure 5:**
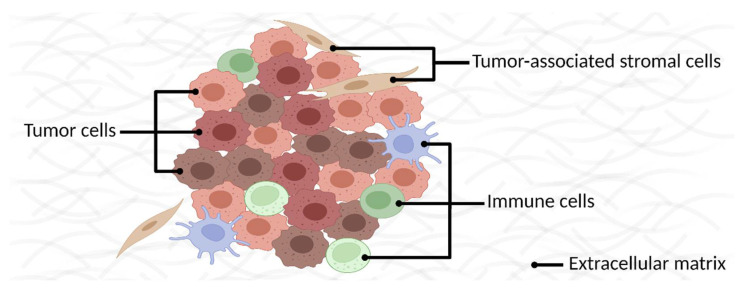
A schematic representation of a generic tumor microenvironment with different cell types.

In the case of when cancer is not caught early enough, membrane protein research also offers unique treatment opportunities. As cancer is a dynamic disease, it becomes more heterogeneous as it progresses. As a result, the bulk tumors comprise a diversity of cell types and their subpopulations with distinct molecular signatures and phenotypes [113] (Figure 5). Furthermore, the same type of tumors harbored in different patients are also dissimilar. This phenotypic heterogeneity plays a crucial role in the variations in treatment effectivity per patient and the ability of tumors to survive a one-fits-all treatment. The conventional treatment with chemotherapy and radiotherapy is non-specific and usually entails a low therapeutic window, provokes adverse effects on off-target cells, and may lead to therapy resistance. However, they are still the primary treatment for many tumors [139]. For an effective cure, it is essential to identify numerous markers, ideally representing the cellular variation in a given tumor and its immediate neighboring cells [43] and then targeting most of them with the drugs.

Again, due to their involvement in all cellular functions, part of the cell-specific molecular signatures and phenotypes within the tumors are mutated or abnormally expressed membrane proteins. Therefore, profiling cell surfaces at different cancer stages would reveal the identity of differentially expressed surface proteoforms. Such information would provide cell-specific, surface-exposed targets for diagnostics, as well as guidance for therapeutic interventions, as much as it would advance the understanding of cancer pathophysiology. For instance, currently, such information would provide invaluable input for the most promising therapy tools at hand, i.e., therapeutic monoclonal antibodies, their derivatives, and continuously evolving antibody-drug conjugates (ADC) for selectively reaching out and killing these cells in the complex tumor microenvironment [140]. Mckertish and Kayser provide a comprehensive list of currently tested and approved ADCs [139] and offer an extensive view of the clinical trials’ results and technical challenges that still need to be optimized. While being very promising, compared to the already accepted tumor markers for diagnostics and drug targets, as shown in Figure 4, more clinical data are required to implement the ADC therapeutics targeting newly discovered membrane proteins into established cancer treatment modalities.

Another high impact of a known membrane proteome on the treatment would be the functional classification of the identified proteins through interactive databases and by identifying the relevant cellular signaling pathways and networks leading to the phenotype, hence providing the cellular signaling pathways as drug targets. For instance, in an elegant work, Karcini and Lazar identified the cell surface proteome of a metastatic breast cancer cell line (SKBR3) [141]. They showed the full potential of such knowledge—from new insights into the disease mechanism—to match protein targets with currently available or on-trial drugs. The network analysis showed that tumor cells’ aberrant proliferation and metastatic potential resulted from the concerted actions of membrane proteins on the signaling pathways together with their functional role on the membrane. Several membrane proteins with newly realized activities in oncogenesis were identified, including nectins, ephrins, bone morphogenic proteins, and families of metalloproteases. Data also revealed that not just the cluster of differentiation (CD) antigens but all cell membrane protein categories contributed to the immune responses, providing numerous immunological markers for exploration.

In conclusion, we believe that the technical developments for the enrichment and identification of membrane proteins, in combination with a growing number of open databases and enhanced data analysis tools, will generate more in-depth knowledge of the biology of the disease at any stage up to matching drugs to the targets for more effective and personalized diagnoses and treatment. Soon, it will be possible to even predict the progress of cancer right at the beginning of the process via the combination of target molecules and complementing attributes.

## Data Availability

Not applicable.
